# (*E*)-*N*′-(5-Bromo-2-hy­droxy­benzyl­idene)-3-meth­oxy­benzohydrazide

**DOI:** 10.1107/S1600536810024001

**Published:** 2010-06-26

**Authors:** Shi-Yong Liu, Xiaoling Wang

**Affiliations:** a College of Chemistry & Pharmacy, Taizhou University, Taizhou Zhejiang 317000, People’s Republic of China; b Department of Chemistry, Liaoning Normal University, Dalian 116029, People’s Republic of China

## Abstract

In the title compound, C_15_H_13_BrN_2_O_3_, the two benzene rings form a dihedral angle of 16.9 (2)°. An intra­molecular O—H⋯N hydrogen bond affects the mol­ecular conformation. In the crystal structure, mol­ecules are linked through N—H⋯O hydrogen bonds into chains running along the *a* axis.

## Related literature

For the medicinal applications of hydrazone compounds, see: Hillmer *et al.* (2010 ▶); Zhu *et al.* (2009 ▶); Jimenez-Pulido *et al.* (2008 ▶); Raj *et al.* (2007 ▶); Zhong *et al.* (2007 ▶). For hydrazones we have reported previously, see: Liu & You (2010*a* ▶,*b* ▶,*c* ▶). For the structures of similar hydrazone compounds, see: Khaledi *et al.* (2009 ▶); Warad *et al.* (2009 ▶); Back *et al.* (2009 ▶); Vijayakumar *et al.* (2009 ▶). For related structures, see: Cao (2009 ▶); Xu *et al.* (2009 ▶); Shafiq *et al.* (2009 ▶).
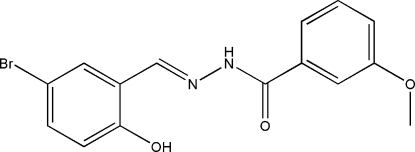


## Experimental

### 

#### Crystal data

C_15_H_13_BrN_2_O_3_
*M* *_r_* = 349.18
Monoclinic, 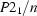

*a* = 6.865 (2) Å
*b* = 30.726 (3) Å
*c* = 7.257 (2) Å
β = 104.437 (15)°
*V* = 1482.2 (7) Å^3^
*Z* = 4
Mo *K*α radiation
μ = 2.78 mm^−1^
*T* = 298 K
0.27 × 0.25 × 0.23 mm

#### Data collection

Bruker SMART CCD area-detector diffractometer
Absorption correction: multi-scan (*SADABS*; Bruker, 2001 ▶) *T*_min_ = 0.520, *T*_max_ = 0.567
8593 measured reflections
3079 independent reflections
1832 reflections with *I* > 2σ(*I*)
*R*_int_ = 0.033

#### Refinement

*R*[*F* ^2^ > 2σ(*F* ^2^)] = 0.035
*wR*(*F* ^2^) = 0.093
*S* = 1.01
3079 reflections
195 parameters
1 restraint
H atoms treated by a mixture of independent and constrained refinement
Δρ_max_ = 0.25 e Å^−3^
Δρ_min_ = −0.45 e Å^−3^

### 

Data collection: *SMART* (Bruker, 2007 ▶); cell refinement: *SAINT* (Bruker, 2007 ▶); data reduction: *SAINT*; program(s) used to solve structure: *SHELXTL* (Sheldrick, 2008 ▶); program(s) used to refine structure: *SHELXTL*; molecular graphics: *SHELXTL*; software used to prepare material for publication: *SHELXTL*.

## Supplementary Material

Crystal structure: contains datablocks global, I. DOI: 10.1107/S1600536810024001/sj5025sup1.cif

Structure factors: contains datablocks I. DOI: 10.1107/S1600536810024001/sj5025Isup2.hkl

Additional supplementary materials:  crystallographic information; 3D view; checkCIF report

## Figures and Tables

**Table 1:** Hydrogen-bond geometry (Å, °)

*D*—H⋯*A*	*D*—H	H⋯*A*	*D*⋯*A*	*D*—H⋯*A*
O1—H1⋯N1	0.82	1.90	2.625 (3)	146
N2—H2⋯O2^i^	0.90 (1)	1.98 (1)	2.852 (3)	163 (3)

Symmetry code: (i) 
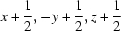
.

**Fig. 1.**
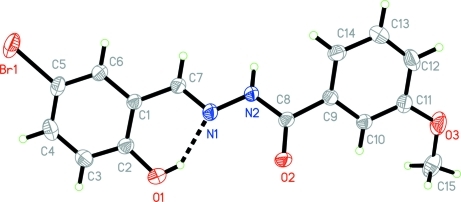
The molecular structure of the title compound. Displacement ellipsoids are drawn at the 30% probability level. Hydrogen atoms are shown as spheres of arbitrary radius and the intramolecular hydrogen bond is drawn as a dashed line.

**Fig. 2.**
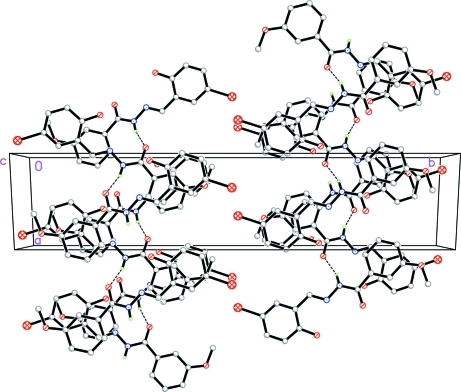
The molecular packing of the title compound, viewed along the c axis. Hydrogen bonds are shown as dashed lines. Hydrogen atoms not involved in hydrogen bonding have been omitted.

## supplementary crystallographic information

### Comment

Considerable attention has been focused on hydrazones and their medicinal applications (Hillmer *et al.*, 2010; Zhu *et al.*, 2009; Jimenez-Pulido *et al.*, 2008; Raj *et al.*, 2007; Zhong *et al.*, 2007). The study on the crystal structures of such compounds is of particular interest (Khaledi *et al.*, 2009; Warad *et al.*, 2009; Back *et al.*, 2009; Vijayakumar *et al.*, 2009). As a continuation of our work on such compounds (Liu & You, 2010*a*,b,c), we report herein the crystal structure of the title compound a new hydrazone.

The molecular structure of the title compound is shown in Fig. 1. The dihedral angle between the C1—C6 and C9—C14 benzene rings is 16.9 (2)°. All the bond lengths are comparable to those observed in related structures (Cao, 2009; Xu *et al.*, 2009; Shafiq *et al.*, 2009) and those we reported previously.

In the crystal structure, molecules are linked through N—H···O hydrogen bonds, to form one-dimensional chains running along the *a* axis (Fig. 2 and Table 1).

### Experimental

The title compound was prepared by the condensation reaction of 5-bromosalicylaldehyde (0.05 mol, 10 g) and 3-methoxybenzohydrazide (0.05 mol, 8.3 g) in anhydrous methanol (200 ml) at ambient temperature. Colourless block-shaped single crystals suitable for X-ray structural determination were obtained by slow evaporation of the solution for a period of a week.

### Refinement

H2 was located from a difference Fourier map and refined isotropically, with the N–H distance restrained to 0.90 (1) Å. The remaining H atoms were positioned geometrically and constrained to ride on their parent atoms, with C–H distances of 0.93–0.96 Å, O–H distance of 0.82 Å, and with *U*_iso_(H) = 1.2*U*_eq_(C) and 1.5*U*_eq_(O and C_methyl_).

### Crystal data

**Table:**

C_15_H_13_BrN_2_O_3_	*F*(000) = 704
*M**_r_* = 349.18	*D*_x_ = 1.565 Mg m^−^^3^
Monoclinic, *P*2_1_/*n*	Mo *K*α radiation, λ = 0.71073 Å
Hall symbol: -P 2yn	Cell parameters from 2100 reflections
*a* = 6.865 (2) Å	θ = 2.6–25.0°
*b* = 30.726 (3) Å	µ = 2.78 mm^−^^1^
*c* = 7.257 (2) Å	*T* = 298 K
β = 104.437 (15)°	Block, colourless
*V* = 1482.2 (7) Å^3^	0.27 × 0.25 × 0.23 mm
*Z* = 4	

### Data collection

**Table:**

Bruker SMART CCD area-detector diffractometer	3079 independent reflections
Radiation source: fine-focus sealed tube	1832 reflections with *I* > 2σ(*I*)
graphite	*R*_int_ = 0.033
ω scans	θ_max_ = 26.8°, θ_min_ = 1.3°
Absorption correction: multi-scan (*SADABS*; Bruker, 2001)	*h* = −5→8
*T*_min_ = 0.520, *T*_max_ = 0.567	*k* = −37→38
8593 measured reflections	*l* = −9→9

### Refinement

**Table:**

Refinement on *F*^2^	Primary atom site location: structure-invariant direct methods
Least-squares matrix: full	Secondary atom site location: difference Fourier map
*R*[*F*^2^ > 2σ(*F*^2^)] = 0.035	Hydrogen site location: inferred from neighbouring sites
*wR*(*F*^2^) = 0.093	H atoms treated by a mixture of independent and constrained refinement
*S* = 1.01	*w* = 1/[σ^2^(*F*_o_^2^) + (0.0443*P*)^2^] where *P* = (*F*_o_^2^ + 2*F*_c_^2^)/3
3079 reflections	(Δ/σ)_max_ = 0.001
195 parameters	Δρ_max_ = 0.25 e Å^−^^3^
1 restraint	Δρ_min_ = −0.45 e Å^−^^3^

### Special details

**Table:**

Geometry. All e.s.d.'s (except the e.s.d. in the dihedral angle between two l.s. planes) are estimated using the full covariance matrix. The cell e.s.d.'s are taken into account individually in the estimation of e.s.d.'s in distances, angles and torsion angles; correlations between e.s.d.'s in cell parameters are only used when they are defined by crystal symmetry. An approximate (isotropic) treatment of cell e.s.d.'s is used for estimating e.s.d.'s involving l.s. planes.
Refinement. Refinement of *F*^2^ against ALL reflections. The weighted *R*-factor *wR* and goodness of fit *S* are based on *F*^2^, conventional *R*-factors *R* are based on *F*, with *F* set to zero for negative *F*^2^. The threshold expression of *F*^2^ > σ(*F*^2^) is used only for calculating *R*-factors(gt) *etc*. and is not relevant to the choice of reflections for refinement. *R*-factors based on *F*^2^ are statistically about twice as large as those based on *F*, and *R*- factors based on ALL data will be even larger.

### Fractional atomic coordinates and isotropic or equivalent isotropic displacement parameters (Å^2^)

**Table:**

	*x*	*y*	*z*	*U*_iso_*/*U*_eq_	
Br1	−0.14621 (6)	0.006913 (10)	0.72146 (5)	0.08082 (18)	
N1	−0.0463 (3)	0.21192 (6)	0.5786 (3)	0.0474 (5)	
N2	0.1078 (3)	0.24188 (7)	0.6071 (3)	0.0486 (6)	
O1	−0.4272 (3)	0.18973 (6)	0.5066 (3)	0.0620 (5)	
H1	−0.3314	0.2060	0.5117	0.093*	
O2	−0.0895 (3)	0.29146 (5)	0.4185 (3)	0.0522 (5)	
O3	0.3228 (3)	0.43040 (6)	0.6485 (3)	0.0755 (6)	
C1	−0.1528 (4)	0.14004 (8)	0.6243 (3)	0.0415 (6)	
C2	−0.3592 (4)	0.14938 (8)	0.5621 (4)	0.0458 (6)	
C3	−0.4970 (4)	0.11647 (10)	0.5578 (4)	0.0568 (7)	
H3	−0.6336	0.1229	0.5227	0.068*	
C4	−0.4370 (5)	0.07446 (9)	0.6041 (4)	0.0599 (8)	
H4	−0.5324	0.0526	0.5971	0.072*	
C5	−0.2338 (5)	0.06462 (8)	0.6614 (4)	0.0524 (7)	
C6	−0.0948 (4)	0.09725 (8)	0.6719 (4)	0.0481 (7)	
H6	0.0414	0.0906	0.7119	0.058*	
C7	0.0005 (4)	0.17332 (8)	0.6411 (4)	0.0457 (7)	
H7	0.1341	0.1666	0.6979	0.055*	
C8	0.0728 (4)	0.28138 (8)	0.5248 (4)	0.0419 (6)	
C9	0.2439 (4)	0.31261 (8)	0.5746 (3)	0.0400 (6)	
C10	0.1979 (4)	0.35642 (8)	0.5838 (3)	0.0423 (6)	
H10	0.0642	0.3652	0.5579	0.051*	
C11	0.3486 (4)	0.38683 (8)	0.6308 (4)	0.0502 (7)	
C12	0.5471 (5)	0.37320 (10)	0.6659 (4)	0.0631 (8)	
H12	0.6500	0.3936	0.6975	0.076*	
C13	0.5937 (4)	0.33037 (10)	0.6550 (4)	0.0621 (8)	
H13	0.7276	0.3219	0.6773	0.074*	
C14	0.4423 (4)	0.29916 (9)	0.6106 (4)	0.0507 (7)	
H14	0.4738	0.2698	0.6053	0.061*	
C15	0.1241 (6)	0.44666 (9)	0.6235 (5)	0.0788 (10)	
H15A	0.0484	0.4413	0.4953	0.118*	
H15B	0.1290	0.4774	0.6477	0.118*	
H15C	0.0605	0.4323	0.7106	0.118*	
H2	0.218 (3)	0.2361 (10)	0.700 (3)	0.080*	

### Atomic displacement parameters (Å^2^)

**Table:**

	*U*^11^	*U*^22^	*U*^33^	*U*^12^	*U*^13^	*U*^23^
Br1	0.1138 (3)	0.03462 (19)	0.0917 (3)	−0.00088 (16)	0.0212 (2)	0.00419 (16)
N1	0.0508 (13)	0.0366 (12)	0.0480 (13)	−0.0043 (10)	−0.0004 (11)	−0.0010 (10)
N2	0.0427 (14)	0.0343 (12)	0.0584 (16)	−0.0037 (10)	−0.0071 (11)	0.0051 (10)
O1	0.0508 (11)	0.0485 (12)	0.0798 (15)	0.0094 (9)	0.0034 (11)	0.0064 (10)
O2	0.0459 (11)	0.0399 (10)	0.0576 (12)	0.0025 (8)	−0.0118 (9)	0.0016 (9)
O3	0.0952 (17)	0.0360 (12)	0.0915 (17)	−0.0141 (11)	0.0163 (13)	−0.0061 (10)
C1	0.0455 (17)	0.0356 (14)	0.0398 (15)	0.0018 (11)	0.0036 (12)	−0.0031 (11)
C2	0.0503 (18)	0.0417 (15)	0.0416 (15)	0.0045 (13)	0.0044 (13)	−0.0005 (12)
C3	0.0486 (18)	0.059 (2)	0.0592 (19)	−0.0049 (14)	0.0069 (15)	0.0009 (15)
C4	0.068 (2)	0.0556 (19)	0.0536 (19)	−0.0215 (15)	0.0104 (16)	−0.0057 (14)
C5	0.074 (2)	0.0326 (14)	0.0478 (17)	−0.0020 (13)	0.0092 (15)	−0.0011 (12)
C6	0.0494 (17)	0.0400 (15)	0.0506 (17)	0.0037 (12)	0.0045 (13)	0.0006 (12)
C7	0.0455 (17)	0.0367 (15)	0.0493 (17)	0.0011 (12)	0.0014 (13)	−0.0012 (12)
C8	0.0438 (16)	0.0340 (14)	0.0431 (16)	0.0030 (12)	0.0016 (13)	−0.0029 (11)
C9	0.0407 (16)	0.0384 (14)	0.0380 (14)	−0.0012 (11)	0.0042 (12)	−0.0005 (11)
C10	0.0421 (15)	0.0398 (15)	0.0420 (15)	0.0006 (11)	0.0049 (12)	0.0016 (11)
C11	0.059 (2)	0.0419 (16)	0.0476 (17)	−0.0059 (13)	0.0095 (14)	0.0020 (13)
C12	0.059 (2)	0.063 (2)	0.063 (2)	−0.0242 (16)	0.0088 (16)	−0.0016 (16)
C13	0.0425 (18)	0.069 (2)	0.072 (2)	−0.0037 (15)	0.0091 (15)	0.0009 (17)
C14	0.0472 (17)	0.0466 (16)	0.0550 (18)	0.0078 (13)	0.0069 (14)	0.0016 (13)
C15	0.108 (3)	0.0404 (18)	0.092 (3)	0.0104 (18)	0.032 (2)	−0.0006 (16)

### Geometric parameters (Å, °)

**Table:**

Br1—C5	1.888 (3)	C5—C6	1.373 (4)
N1—C7	1.282 (3)	C6—H6	0.9300
N1—N2	1.379 (3)	C7—H7	0.9300
N2—C8	1.347 (3)	C8—C9	1.490 (3)
N2—H2	0.898 (10)	C9—C14	1.384 (3)
O1—C2	1.350 (3)	C9—C10	1.388 (3)
O1—H1	0.8200	C10—C11	1.373 (3)
O2—C8	1.226 (3)	C10—H10	0.9300
O3—C11	1.361 (3)	C11—C12	1.387 (4)
O3—C15	1.421 (4)	C12—C13	1.361 (4)
C1—C6	1.392 (3)	C12—H12	0.9300
C1—C2	1.405 (3)	C13—C14	1.392 (4)
C1—C7	1.451 (3)	C13—H13	0.9300
C2—C3	1.380 (4)	C14—H14	0.9300
C3—C4	1.371 (4)	C15—H15A	0.9600
C3—H3	0.9300	C15—H15B	0.9600
C4—C5	1.386 (4)	C15—H15C	0.9600
C4—H4	0.9300		
			
C7—N1—N2	116.7 (2)	O2—C8—N2	122.7 (2)
C8—N2—N1	119.3 (2)	O2—C8—C9	121.9 (2)
C8—N2—H2	122.3 (19)	N2—C8—C9	115.5 (2)
N1—N2—H2	117.0 (19)	C14—C9—C10	120.3 (2)
C2—O1—H1	109.5	C14—C9—C8	122.2 (2)
C11—O3—C15	118.5 (2)	C10—C9—C8	117.5 (2)
C6—C1—C2	118.4 (2)	C11—C10—C9	120.4 (2)
C6—C1—C7	119.2 (2)	C11—C10—H10	119.8
C2—C1—C7	122.4 (2)	C9—C10—H10	119.8
O1—C2—C3	118.8 (2)	O3—C11—C10	125.8 (3)
O1—C2—C1	122.0 (2)	O3—C11—C12	115.1 (2)
C3—C2—C1	119.2 (2)	C10—C11—C12	119.0 (3)
C4—C3—C2	121.5 (3)	C13—C12—C11	121.0 (3)
C4—C3—H3	119.3	C13—C12—H12	119.5
C2—C3—H3	119.3	C11—C12—H12	119.5
C3—C4—C5	119.8 (3)	C12—C13—C14	120.5 (3)
C3—C4—H4	120.1	C12—C13—H13	119.8
C5—C4—H4	120.1	C14—C13—H13	119.8
C6—C5—C4	119.4 (3)	C9—C14—C13	118.7 (3)
C6—C5—Br1	119.7 (2)	C9—C14—H14	120.6
C4—C5—Br1	120.9 (2)	C13—C14—H14	120.6
C5—C6—C1	121.6 (3)	O3—C15—H15A	109.5
C5—C6—H6	119.2	O3—C15—H15B	109.5
C1—C6—H6	119.2	H15A—C15—H15B	109.5
N1—C7—C1	120.6 (2)	O3—C15—H15C	109.5
N1—C7—H7	119.7	H15A—C15—H15C	109.5
C1—C7—H7	119.7	H15B—C15—H15C	109.5

### Hydrogen-bond geometry (Å, °)

**Table:**

*D*—H···*A*	*D*—H	H···*A*	*D*···*A*	*D*—H···*A*
O1—H1···N1	0.82	1.90	2.625 (3)	146
N2—H2···O2^i^	0.90 (1)	1.98 (1)	2.852 (3)	163 (3)

Symmetry codes: (i) *x*+1/2, −*y*+1/2, *z*+1/2.
